# Facilitatory effects of anti-spastic medication on robotic locomotor training in people with chronic incomplete spinal cord injury

**DOI:** 10.1186/s12984-015-0018-4

**Published:** 2015-03-20

**Authors:** Lynsey D Duffell, Geoffrey L Brown, Mehdi M Mirbagheri

**Affiliations:** Department of Physical Medicine and Rehabilitation, Northwestern University, 345 E Superior Street, Chicago, USA; Sensory Motor Performance Program, Rehabilitation Institute of Chicago, 345 E Superior Street, Chicago, USA

**Keywords:** Gait, Muscle, Rehabilitation, Spasticity, Spinal Cord Injury, Hypertonia, Tizanidine, Locomotor, Kinematics, Robotics

## Abstract

**Background:**

The objective of this study was to investigate whether an anti-spasticity medication can facilitate the effects of robotic locomotor treadmill training (LTT) to improve gait function in people with incomplete spinal cord injury (SCI).

**Methods:**

Individuals with chronic incomplete SCI were recruited and carried out a 4 week intervention of either locomotor treadmill training (LTT) alone (n = 26) or LTT combined with Tizanidine (TizLTT), an anti-spasticity medication (n = 22). Gait function was evaluated using clinical outcome measures of gait, speed and endurance. To better understand the underlying mechanisms of the therapeutic effects, maximal strength, active range of motion (AROM) and peak velocity (Vp) of ankle dorsi- and planter-flexor muscles were also measured. Differences were assessed using two-way mixed design analysis of variance. The number of subjects that achieved the minimal important difference (MID) for clinical scores was also measured for each group, and the results of those that did attain the MID were compared with those that did not.

**Results:**

Both LTT and TizLTT resulted in significant improvements in walking speed and dorsiflexion maximum strength, with no significant differences between them, using group-averaging analysis. However, using the MID analysis, a higher proportion of subjects in the TizLTT group achieved the MID for walking speed (40%) compared with LTT alone (13%). Those that achieved the MID for walking speed were significantly higher functioning at baseline than those that did not in the TizLTT group, and the change in walking speed was associated with the change in dorsiflexion peak velocity (R^2^ = 0.40; P < 0.05).

**Conclusion:**

Tizanidine appears to facilitate the effects of LTT on gait function in individuals with chronic SCI that are higher functioning at baseline. We speculate that this may be due to restoration of inhibitory mechanisms by Tizanidine, resulting in greater stretch in the planterflexor muscles during the LTT.

## Background

An incomplete spinal cord injury (SCI) results in the partial loss of motor and sensory function below the level of the injury. Therefore affected individuals retain functional ability to varying degrees. SCI rehabilitation aims to optimize functional recovery after incomplete SCI. One secondary consequence of SCI is neuromuscular abnormalities resulting in hypertonia of muscle groups, which are thought to be the result of a combination of intrinsic and reflex-mediated muscle stiffness [1]. Spasticity, defined as a velocity-dependent resistance to stretch (reflex-mediated hypertonia), is associated with substantially increased muscle activity (measured by electromyography (EMG)) during stretch compared with healthy subjects under passive conditions [2,3], which may be due to reduced inhibitory mechanisms in chronic SCI subjects [4,5].

Hypertonia commonly affects the muscles surrounding the ankle joint, which have important roles during functional tasks [6]; however the relationship between hypertonia and gait function remains controversial. It has been suggested that the reduced inhibitory mechanisms observed in spastic muscles under passive conditions do not further decrease during voluntary contractions [7]. In healthy subjects inhibitory mechanisms do decrease from passive to voluntary conditions, resulting in similar levels of inhibition during voluntary contractions between healthy people and patients with spasticity [7]. In support, clinical indications of spasticity (measured in the passive muscle) have been proposed to be unrelated to gait impairment, as evidenced by preserved timing of muscle activation during spastic gait in stroke and SCI patients [7,8]. Additionally, based on clinical observations, reduced hypertonia was not associated with improved function [9,10]. Conversely, our earlier study noted increased reflex stiffness in SCI compared with healthy subjects under active conditions [11] and other studies have demonstrated that increased hypertonia relates to impaired function [12,13].

Tizanidine, an anti-spasticity medication, has been shown to reduce hypertonia in SCI individuals, evidenced by a reduction in passive resistance [14-17]. As an α2 noradrenergic agonist, it is thought to reduce hypertonia through depression of dorsal horn interneuron excitability [18], thereby reducing reflex-mediated hypertonia (spasticity). The effects of Tizanidine on functional outcomes have seldom been studied [19], thus the effects of Tizanidine on gait impairment remain ambiguous. It has been reported that Tizanidine significantly improved voluntary activation (muscle activity) [15] and substantially reduced reflex mechanical responses [20] in SCI individuals. In addition, Tizanidine has been shown to facilitate locomotor capacity in spinalized cats [21]. Thus Tizanidine may improve gait function through several mechanisms including restored inhibitory mechanisms and improved voluntary activation.

A popular tool intended to optimize recovery of gait function specifically after SCI is locomotor treadmill training (LTT) [22], which may incorporate body-weight supported and/or robotic-assistive gait training. Previous studies have reported improvements in overground walking speed [23] [24] and endurance [23] after LTT in people with chronic SCI. LTT has also been reported to reduce abnormal neuromuscular activity, measured by clinical scores [25], EMG activity [26,27], or neuromuscular mechanical properties [28], although these changes did not correlate with functional improvements [25]. A recent review article however suggested that the evidence for LTT is limited [29] since studies often omit alternative intervention groups, and those that do include alternative interventions have found similar improvements from conventional physical therapy and overground walking training compared with LTT [24,30,31].

Recently therefore, combination therapies have been proposed in an attempt to surpass the outcomes of single interventions [32]. Some evidence in support of this theory comes from combined functional electrical stimulation (FES) and LTT studies, which have noted improved gait quality when using combined FES + LTT and FES + overground walking therapies than robotic locomotor training [24] or regular physical therapy [33] provided alone. In SCI patients, the potential for functional improvements from LTT may be limited by prevailing spasticity. We recently demonstrated that Tizanidine improved walking speed and endurance in some patients that were higher functioning [34]. Tizanidine may have allowed greater voluntary activation and/or range of motion at the ankle joint and may therefore facilitate LTT resulting in greater improvements in both walking speed and endurance. The combined effects of anti-spasticity medication and LTT in people with incomplete SCI have seldom been investigated. One study found improvements in walking speed and endurance in two case studies; these individuals were taking baclofen, as part of their usual care, during an intervention of LTT. These individuals were in the acute phase of injury and there was no control comparison, therefore the facilitatory effects of the anti-spasticity medication could not be separated from that of the LTT and spontaneous recovery [35]. The facilitatory effects of Tizanidine on LTT have not previously been reported, except in our interim conference reports [36-38].

Importantly, the population of people living long-term with chronic incomplete SCI is heterogeneous in terms of functional levels, and the previously reported extents and rates of improvement in functional performance from interventions are variable [29]. We recently demonstrated that group averaging techniques can therefore mask important data. We reported that both Tizanidine and LTT interventions resulted in improvements in walking speed and endurance, with no difference between interventions. However, we further analyzed the data of individuals that achieved improvements greater than the minimal important difference (MID) for a given clinical test, and noted that Tizanidine administered alone was more beneficial for higher functioning individuals. Therefore, MID analysis should be employed alongside group-averaging techniques to further explore the effects of interventions, in such heterogeneous populations.

This study aimed to address two questions: i) can Tizanidine combined with LTT improve clinical gait scores in people with incomplete SCI to a greater extent than LTT alone and ii) if Tizanidine does facilitate LTT, what are the underlying mechanisms. We used a combination of analysis techniques, including group averaging and MID to answer these questions. We hypothesized that LTT would improve walking speed when administered alone, and that Tizanidine would facilitate LTT in higher functioning subjects, resulting in greater improvements in walking speed compared with LTT alone. In order to explore the underlying mechanisms associated with functional outcomes, we investigated the effects of LTT alone and Tizanidine combined with LTT on muscle strength of ankle plantar- and dorsi-flexor muscles, active range of motion and peak movement velocity of dorsiflexors.

## Methods

Subjects with either cervical or thoracic incomplete spinal cord injury, as a result of trauma, were recruited from the outpatient service at the Rehabilitation Institute of Chicago. All subjects provided written informed consent and the study had ethical approval from the Northwestern University Institutional Review Board. Inclusion criteria were aged >18 years, motor incomplete SCI (ASIA Impairment Scale [AIS] classification C or D) with level of injury above T10 and >12 months post injury, ambulatory or potentially ambulatory, medical clearance to participate, evidence of clinical spasticity in the muscles surrounding the ankle and knee joints (Modified Ashworth Score (MAS) ≥1), and lower-limb passive range of motion within functional limits for ambulation. Exclusion criteria were sitting tolerance <2 hours, existing infection, severe cardiovascular or pulmonary disease, concomitant neurological injury, history of fractures post-SCI, and known orthopedic or peripheral nerve injury in the lower extremities.

Subjects were randomly assigned into one of two intervention groups; LTT alone (LTT; n = 26) or combined LTT and Tizanidine (TizLTT; n = 22). Subjects assigned to the TizLTT group, were initially provided with Tizanidine alone for a period of 4 weeks, and results for that period have been presented elsewhere, together with the LTT group clinical outcomes [34]. Twenty seven subjects in total were recruited into the TizLTT group, however only 22 subjects continued to the combined treatment. Subject characteristics for the two groups are provided in Table 1.

**Table 1:** **Mean (SD) characteristics of subjects in the LTT alone (LTT) and combined LTT and Tizanidine (TizLTT) groups**

	**LTT (n = 26)**	**TizLTT (n = 22)**
Gender	7 F; 19 M	7 F; 15 M
Age (years)	46.6 (12.6)	46.5 (11.9)
Time since injury (years)	9.3 (8.9)	10.2 (10.4)
Level of injury	20 C; 6 T	13 C; 9 T
WISCI II score	14.7 (5.2)	15.2 (5.1)

F = Female; M = Male; C = Cervical; T = Thoracic; WISCII II = Walking Index for Spinal Cord Injury II.

### Interventions

Locomotor training was provided using a robot-assisted locomotor training device (Lokomat, Hocoma AG, Switzerland). This device provides body-weight supported gait assistance such that the individual is suspended in a harness over a motorized treadmill while the frame of the robot, attached by straps to the outside of the lower limbs, moves the limbs in a natural walking pattern.

Training was provided three times per week for four weeks; each session lasted ≤1 hour, with 30–45 minutes of training. Treadmill speed, body-weight support, and robotic guidance forces were determined by the physical therapist, based on tolerance and comfort of the subject. Generally however, reducing guidance force was prioritized to promote voluntary drive to muscles, and to minimize passive training. Participants were encouraged to contribute to the gait training as much as possible and to increase gait speed. Body-weight support was configured to maximize lower-extremity loading without producing excessive knee flexion during the stance phase, or allowing toe-drag during the swing phase. Subjects were instructed to “walk with the robot” to ensure that the lower-extremity movements were consistent with the Lokomat stepping pattern. Subjects were also instructed to pay attention to their ankle movements during the gait cycle i.e. to focus on planting the heel of their foot at heel-strike and to “lift their toes” during the swing phase. A mirror placed in front of the subjects provided visual feedback.

For the TizLTT group, .03 mg/kg of Tizanidine was administered four times a day for eight weeks (with LTT provided during the final 4 weeks). This dosage represents a useful compromise, in that it usually shows efficacy [20], but does not cause overwhelming side effects. In the first week, administration of the drug was progressively increased until the full dosage was received on day 7, and the full dosage was then administered for a subsequent 8-week period. Subjects taking muscle relaxant medications prior to enrolling on the study were tapered from their medication prior to the start of the study.

### Outcome measures

Outcomes were measured at 0, 1, 2 and 4 weeks from the start of LTT for both groups. Outcome measures were: i) the Timed up and go (TUG) whereby subjects are instructed to stand up from an armed chair, walk 3 meters, turn, return to the chair and sit down; ii) the 10-meter walk test (10MWT) performed at the fastest speed, whereby subjects are instructed to walk 10 meters as quickly and safely as possible and; iii) the 6-minute walk test (6MWT) whereby subjects are instructed to walk for 6 minutes and the distance covered is measured. Functional measures were chosen to evaluate different aspects of gait impairment including mobility and balance, walking speed and endurance, respectively.

For voluntary activation measures, subjects were seated in a custom-built isokinetic dynamometer. The ankle of their most spastic side (assessed by the MAS) was strapped to a footplate, which was attached to the rotational axis of a servomotor, and their knee was fixed at an angle of 150°. Ankle joint angular position and torque were measured by a rotary encoder and a 6-axis torque transducer, respectively. Data were sampled at 1 kHz by a 16 bit A/D converter, and anti-alias filtered on-line at 200 Hz. Subjects were instructed to push down and pull up with their foot “as hard and as fast as possible” to assess maximal isometric muscle strength of plantar- and dorsi-flexor muscles, respectively, with the ankle fixed at an angle of 90° (maximum voluntary isometric contractions; MVIC). Subjects also carried out maximal isokinetic voluntary contractions by rotating the foot from maximum plantarflexion to maximum dorsiflexion (when the motor was deactivated) to assess active range of motion (AROM) and peak isokinetic velocity (Vp). Isometric and isokinetic tests were both repeated twice; the MVIC with the highest plateau force (isometric) and the trial with the highest ROM (isokinetic) were used for analysis.

### Data analysis

Maximal isometric torque during both plantar- and dorsi-flexion MVICs was measured as the average torque over the 3-second period in which the torque standard deviation was lowest. For isokinetic contractions, Vp was measured as described previously [39]. Briefly, the onset of the task was defined as the first sample with an acceleration >5% peak acceleration. The end of the task was defined as the final sample with an acceleration >5% peak acceleration. AROM was taken as the difference between the angular position of the ankle at the onset and end of the task (as defined above). Vp was taken as the peak measured velocity between the onset and end of the task.

Independent t-tests were used to compare subject characteristics and baseline clinical scores between groups. Fisher’s exact tests were used to compare the gender and injury level (cervical *vs* thoracic) distribution between groups. Two way mixed design analysis of variance (ANOVA) was used to identify significant changes due to time-point (within-subject) and group (between-subject). MID values were calculated using the formula proposed by Beckerman et al. [40] (1.96*√2*SEM) based on our previously presented control data. The MID values were 0.11 m/s, 37.1 m, and −14.5 s for the 10MWT, 6MWT and TUG, respectively [34]. The number of subjects that achieved these MID values were calculated. In order to determine the characteristics of the subjects that achieved the MID, WISCI II and baseline scores for each measure were compared between those that did and did not achieve the MID, using independent t-tests. Finally, changes in MVIC torque, AROM and peak movement velocity were compared using two way ANOVAs to better understand the underling mechanisms of the change in those that did achieve the MID. An alpha value <0.05 was considered significant.

## Results

There was no significant difference in age, time since injury and WISCI II scores between the two groups (p > 0.05; Table 1). The distribution of males:females and level of injury (cervical:thoracic) were also not significantly different between groups (p > 0.05; Table 1). Baseline scores for walking speed (10MWT), endurance (6MWT) and TUG were not significantly different between groups (p > 0.05).

### Group averaging analysis (ANOVA)

**Figure 1:**
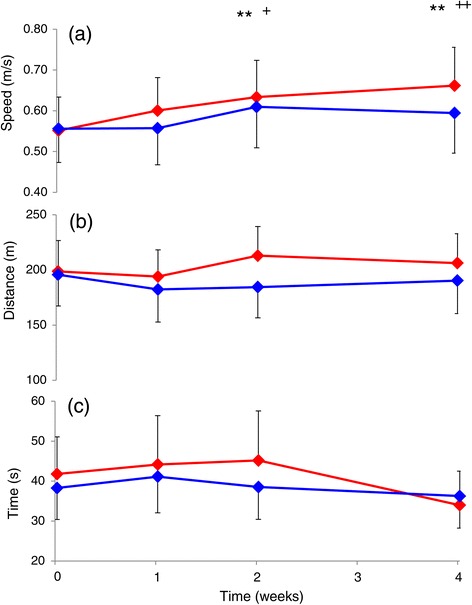
**Mean (SEM) walking (a) speed from 10 minute walk test, (b) distance from the 6-minute walk test and c) time from the timed up and go test for the locomotor treadmill training (LTT; blue) and LTT combined with Tizanidine (TizLTT; red) groups.** **p < 0.001 compared with baseline and ^+^p < 0.003 and ^++^p < 0.001 compared with Week 2.

Walking speed improved significantly with time for both groups (p < 0.001) with no difference between groups (Figure 1a). There were no significant changes with time for the TUG and 6MWT (Figure 1b-c). There was a small but significant increase in maximum dorsiflexion torque after 4 weeks due to LTT or TizLTT (p < 0.05), with no significant difference between groups (Table 2). There were no significant changes in plantarflexion maximal torque, AROM and Vp with time and between groups (Table 2).

**Table 2:** **Mean (SEM) maximum voluntary isometric contraction (MVIC) plantarflexion (PF) and dorsiflexion (DF) torque, active range of motion (AROM) and peak movement velocity (Vp) for locomotor treadmill training alone (LTT) and LTT combined with Tizanidine (TizLTT) groups at 0, 1, 2 and 4 weeks after the start of LTT**

	**MVIC PF (Nm)**		**MVIC DF (Nm)**		**AROM (°)**		**Vp (°/s)**	
	**LTT**	**TizLTT**	**LTT**	**TizLTT**	**LTT**	**TizLTT**	**LTT**	**TizLTT**
Week 0	28.1 (4.4)	30.8 (4.5)	12.3 (1.4)	11.5 (1.7)	34.1 (3.3)	45.1 (7.5)	117.1 (13.5)	165.1 (26.2)
Week 1	26.2 (3.4)	33.8 (5.8)	12.2 (1.4)	12.0 (2.0)	37.1 (3.1)	48.7 (7.3)	130.7 (14.1)	182.6 (26.8)
Week 2	27.6 (3.9)	33.9 (6.6)	12.0 (1.4)	12.1 (2.0)	36.3 (3.4)	46.6 (7.2)	126.8 (14.6)	179.6 (27.4)
Week 4	28.4 (4.1)	33.7 (5.6)	13.2* (1.7)	13.0* (2.1)	35.6 (3.1)	49.2 (7.4)	121.5 (13.6)	186.9 (28.1)

*Denotes significant difference compared with 0, 1 and 2 weeks (p < 0.05).

### MID analysis

**Figure 2:**
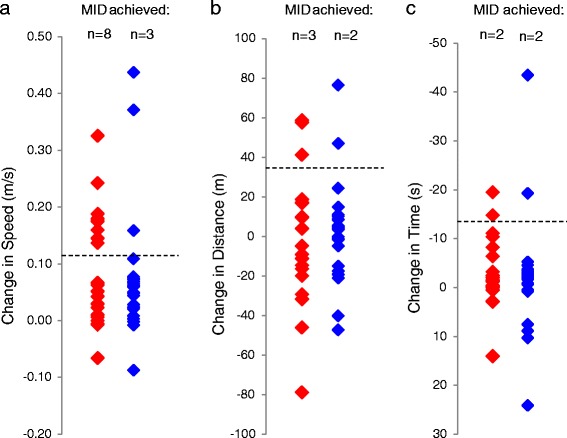
**Change in clinical scores at 4 weeks from baseline for the (a) 10 meter walk test, (b) 6 minute walk test and (c) timed up and go, for subjects in the locomotor treadmill training (LTT; blue) and LTT combined with Tizanidine (TizLTT; red) groups.** Dashed lines denote the minimal important difference (MID) for each test [34].

The number of subjects that achieved the MID for each clinical test in both groups is shown in Figure 2a-c. For the 6MWT and TUG, only 2–3 subjects achieved the MID, irrespective of intervention group. However, for the 10MWT, 8 subjects (40%) in the combined TizLTT group had achieved the MID after 4 weeks of training, compared with only 3 subjects (13%) in the LTT alone group (Figure 2). The subjects that achieved the MID for walking speed were higher functioning individuals at baseline in the TizLTT group (Table 3), evidenced by significantly higher WISCI II scores (p < 0.01), and significantly improved baseline scores for the 10MWT (p = 0.02), 6MWT (p = 0.02) and TUG (p = 0.01). Subjects that achieved the MID for walking speed in the LTT alone group were not higher functioning compared with those that did not, when assessed by WISCI II scores (p > 0.05), however their baseline scores for walking speed (p = 0.01) and endurance (p = 0.02) were significantly higher among the subjects that achieved the MID for walking speed. Baseline MVIC, AROM and Vp did not differ between those that did and did not attain the MID for walking speed in both groups (Table 3). These findings in the LTT group should be interpreted with caution due to the low number of subjects that did achieve the MID for walking speed (n = 3).

**Table 3:** **Mean (SEM) baseline clinical scores and baseline kinetic and kinematic variables for subjects in the combined locomotor treadmill training and Tizanidine (TizLTT) and locomotor treadmill training alone (LTT) groups that did and did not achieve the minimal important difference (MID) for walking speed (10MWT)**

**Group**		**TizLTT**			**LTT**		
**Test**		**MID achieved**	**MID not achieved**	**P-value**	**MID achieved**	**MID not achieved**	**P-value**
WISCI II score		18.9 (0.9)	14.3 (1.1)	<0.01	16.0 (3.5)	14.4 (1.2)	0.70
10MWT speed (m/s)		0.8 (0.1)	0.4 (0.1)	0.02	1.1 (0.3)	0.5 (0.1)	0.01
6MWT distance (m)		280.5 (30.8)	154.6 (35.8)	0.02	364.5 (92.8)	171.6 (28.1)	0.02
TUG time (s)		15.0 (1.9)	51.8 (12.5)	0.01	12.1 (3.1)	45.1 (9.5)	0.18
MVIC torque	PF	30.7 (6.3)	30.9 (6.7)	0.98	46.6 (7.9)	25.4 (3.9)	0.09
	DF	11.4 (2.2)	11.7 (2.9)	0.94	17.1 (2.7)	11.6 (1.5)	0.16
AROM		54.2 (15.2)	40.2 (8.3)	0.55	48.4 (5.8)	32.9 (3.7)	0.09
Vp		189.4 (42.5)	151.9 (34.0)	0.65	160.7 (30.4)	115.4 (15.9)	0.27

Walking index for spinal cord injury II (WISCI II); 10 meter walk test (10MWT); 6 minute walk test (6MWT); timed up and go (TUG); maximum voluntary isometric contraction (MVIC); plantarflexors (PF); dorsiflexors (DF); active range of motion (AROM) and peak velocity during isokinetic dorsiflexion (Vp).

**Figure 3:**
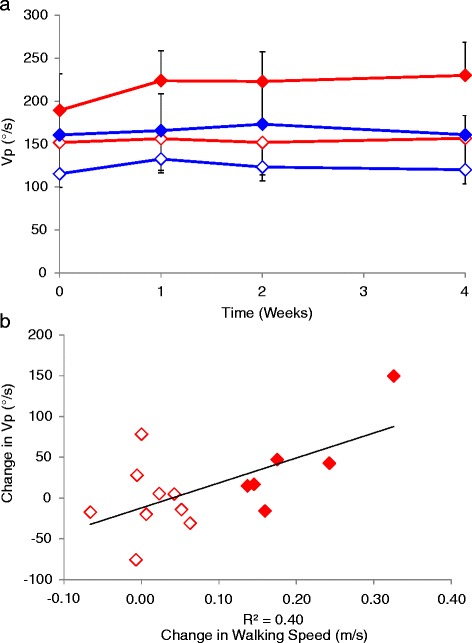
**Peak velocity (Vp) during dorsiflexion in the combined locomotor treadmill training (LTT; blue) and Tizanidine (TizLTT; red) group. (a)** Mean (SEM) at 0, 1, 2 and 4 weeks into the intervention and **(b)** change in Vp plotted against change in walking speed from baseline to the 4th week assessment, for subjects in the TizLTT group that did (filled diamonds) and did not (open diamonds) achieve the minimal important difference (MID) in the 10MWT.

Changes in MVIC, AROM and Vp were compared between the subjects that did and did not achieve the MID, based on the 10MWT only, since the number of subjects that achieved the MID was too low in other groups to make meaningful conclusions. Changes in MVIC, AROM and Vp with time did not differ between the subjects that did and did not achieve the MID for walking speed among both intervention groups, however Vp tended to increase in the subjects that achieved the MID within the TizLTT group compared with those that did not attain the MID, and all subjects in the LTT alone group (Figure 3a). There was also a significant moderate correlation between the change in walking speed and change in Vp among the TizLTT group (R^2^ = 0.40, p < 0.05; Figure 3b).

## Discussion

This study measured the facilitatory effects of Tizanidine, an anti-spasticity medication, on LTT in people with chronic SCI. We have noted that both LTT alone and LTT combined with Tizanidine significantly improved walking speed and maximum dorsiflexion torque. The number of subjects that achieved the MID was highest among the TizLTT group, with 40% of individuals achieving the MID for walking speed. Among that group, the subjects that achieved the MID were significantly higher functioning, and changes may relate to improvements in dorsiflexion peak velocity. These findings indicate that Tizanidine can facilitate the locomotor training in people with SCI that are higher functioning, perhaps by improved ankle dorsiflexion through reduced spasticity in plantarflexors.

Locomotor treadmill training appeared to improve walking speed, supporting our first hypothesis. Improvements in walking speed have been reported previously after LTT [23,24,30,34,41,42], which have been associated with improvements in overall muscle strength and balance [30]. We also noted an associated improvement in dorsiflexion MVIC, however the change was relatively small. Our group-averaging analysis revealed that the addition of Tizanidine, an α2 noradrenergic agonist, to LTT did not increase or decrease the overall magnitude of change, as predicted in our second hypothesis. Tizanidine has been shown to reduce hypertonia in SCI individuals as assessed by a reduction in passive resistance [14-17], however its direct benefit on gait outcomes remain ambiguous. Hypertonia results in exaggerated reflex activity as well as increased intrinsic stiffness in the muscle and surrounding tissue; Tizanidine depresses interneuron excitability [18], thus it attempts to reduce exaggerated reflex activity (spasticity). This may be inadequate to improve gait function since it has been proposed that reflex activity is increased in SCI compared with healthy people only during passive stretch of muscle [7], although our earlier study did note increased reflex stiffness in SCI compared with healthy subjects under active conditions [11].

Alternatively, Tizanidine may enhance gait function in some but not all SCI individuals. We previously reported the effects of a similar dose of Tizanidne alone for 4 weeks on clinical scores, and found that the participants that attained the minimal important difference (a magnitude of change that was greater than the variability in the measurement technique) for both walking speed and endurance were higher functioning individuals, evidenced by significantly higher WISCI II and baseline clinical scores [34]. Thus, Tizanidine may have facilitated LTT in specific individuals, resulting in a higher number of individuals achieving a clinically relevant improvement in function (the MID for walking speed) than LTT alone, as opposed to increasing walking speed to a greater overall magnitude than with LTT alone, as we hypothesized. Indeed, using MID analysis, our data showed that a higher proportion of subjects achieved the MID for walking speed in the combined TizLTT group (40%), compared with LTT alone (13%). This finding supports the notion that Tizanidine did have a facilitatory effect on LTT, in specific individuals with SCI.

When comparing those individuals that achieved the MID for the 10MWT with those that did not, we found that the individuals that achieved the MID were higher functioning at baseline, with significantly higher WISCI II scores and improved baseline scores for all clinical measures taken (Table 3). This supports our previous findings [34] that higher functioning individuals may be more likely to respond to LTT and additionally indicates that Tizanidine may facilitate LTT specifically for higher functioning individuals, in terms of improving functional capacity during gait.

There were no significant differences in the baseline measured kinematic and kinetic variables between the subjects that did and did not achieve the MID for walking speed, among the TizLTT group (Table 3). Thus, while baseline functional levels may be predictive of whether or not Tizanidine would be a beneficial adjunct to rehabilitative therapies, the specific neuromuscular properties measured here do not appear to be predictive of clinical outcomes. There was however a significant moderate correlation between the change in dorsiflexion isokinetic peak velocity and change in walking speed for the subjects in the TizLTT group (r^2^ = 0.40; P < 0.05). In addition, peak velocity tended to improve in those that did achieve the MID for walking speed in the TizLTT group but not in those that achieved the MID in the LTT alone group or among the subjects that did not achieve the MID for both groups (Figure 3a). This result may indicate that Tizanidine facilitated LTT by restoring inhibitory mechanisms, which reduced spasticity in plantarflexor muscles. This may have in turn reduced the inhibitory effects of plantarflexor muscles on dorsiflexors, resulting in improved voluntary activation of the ankle dorsiflexors. After SCI, the inhibitory effects of dorsiflexors on plantarflexors typically reduce or become facilitatory [43,44], which can cause co-contraction. Theoretically, restoration of inhibitory mechanisms would reverse these changes, resulting in reduced co-contraction or joint stiffness, allowing greater stretch in the planterflexor muscles to be achieved during LTT [15]. Since our observations of increased peak velocity were not made during LTT, and inhibition was not assessed in this study, these proposed underlying mechanisms are only speculative, and not fully supported by the data presented here. Previous studies have similarly reported improved functional outcomes from combination therapies compared to LTT therapy provided alone [24] and conventional physical therapy [33]; the data presented here provide further support for the use of combinational therapies.

It should be noted that the dose provided in the present study was relatively low (approx. 8.5 mg/day for an individual of 70 kg body mass). Knutsson et al. [15] noted improved voluntary activation (assessed by EMG activity) in SCI patients following 10 mg/day of Tizanidine [15], but improved gait capacity was only noted in 4 patients that were administered a much higher dose of Tizanidine (32 mg/day). In that study, improved gait capacity was attributed to lowered passive resistance in the plantarflexor muscles, thus allowing greater dorsiflexion during the swing phase of gait [15]. In the present study, both LTT and TizLTT were administered over a relatively short period (4 weeks or 12 LTT sessions). For LTT, the total number of training sessions required to improve walking outcomes > MID is in the range 10–130 sessions [45]. Therefore, providing a higher dose of Tizanidine, or providing interventions for a longer duration, may have increased the magnitude of change in walking speed for the combined intervention compared with LTT alone. Finally, there was no control group (no intervention) in this study; control data was presented in our previous publication [34] and can be used as a comparison with the results presented here.

## Conclusions

Tizanidine appears to facilitate the effects of LTT on gait function in higher functioning individuals with SCI. This was evidenced by improvements in walking speed > MID for a higher number of individuals in the TizLTT compared with LTT group. The change in walking speed was associated with the change in isokinetic peak velocity of dorsiflexion in the TizLTT group, and therefore Tizanidine may have facilitated LTT through restoration of inhibitory mechanisms, resulting in greater stretch in the planter flexor muscles during the LTT.
